# Derivation and Validation of a 4-Level Clinical Pretest Probability Score for Suspected Pulmonary Embolism to Safely Decrease Imaging Testing

**DOI:** 10.1001/jamacardio.2021.0064

**Published:** 2021-03-03

**Authors:** Pierre-Marie Roy, Emilie Friou, Boris Germeau, Delphine Douillet, Jeffrey Allen Kline, Marc Righini, Grégoire Le Gal, Thomas Moumneh, Andrea Penaloza

**Affiliations:** 1 Emergency Department, CHU Angers, Institut Mitovasc UMR (CNRS 6015—INSERM 1083), UNIV Angers, F-CRIN INNOVTE, Angers, France; 2 Emergency Department, CHU Angers, Angers, France; 3 Emergency Department, Cliniques Universitaires Saint Luc, Université Catholique de Louvain, Brussels, Belgium; 4 Department of Emergency Medicine, Indiana University School of Medicine, Indianapolis; 5 Division of Angiology and Hemostasis, Department of Internal Medicine, Faculty of Medicine, Geneva University Hospital, Geneva, Switzerland; 6 Ottawa Hospital Research Institute, The Ottawa Hospital, Division of Hematology, Department of Medicine, University of Ottawa, Ottawa, Ontario, Canada; 7 Emergency Department, Cliniques Universitaires Saint Luc, Université Catholique de Louvain, F-CRIN INNOVTE, Brussels, Belgium

## Abstract

**Importance:**

In patients with suspected pulmonary embolism (PE), overuse of diagnostic imaging is an important point of concern.

**Objective:**

To derive and validate a 4-level pretest probability rule (4-Level Pulmonary Embolism Clinical Probability Score [4PEPS]) that makes it possible to rule out PE solely on clinical criteria and optimized D-dimer measurement to safely decrease imaging testing for suspected PE.

**Design, Setting, and Participants:**

This study included consecutive outpatients suspected of having PE from US and European emergency departments. Individual data from 3 merged management studies (n = 11 114; overall prevalence of PE, 11%) were used for the derivation cohort and internal validation cohort. The external validation cohorts were taken from 2 independent studies, the first with a high PE prevalence (n = 1548; prevalence, 21.5%) and the second with a moderate PE prevalence (n = 1669; prevalence, 11.7%). A prior definition of pretest probability target values to achieve a posttest probability less than 2% was used on the basis of the negative likelihood ratios of D-dimer. Data were collected from January 2003 to April 2016, and data were analyzed from June 2018 to August 2019.

**Main Outcomes and Measures:**

The rate of PE diagnosed during the initial workup or during follow-up and the rate of imaging testing.

**Results:**

Of the 5588 patients in the derivation cohort, 3441 (61.8%) were female, and the mean (SD) age was 52 (18.5) years. The 4PEPS comprises 13 clinical variables scored from −2 to 5. It results in the following strategy: (1) very low probability of PE if 4PEPS is less than 0: PE ruled out without testing; (2) low probability of PE if 4PEPS is 0 to 5: PE ruled out if D-dimer level is less than 1.0 μg/mL; (3) moderate probability of PE if 4PEPS is 6 to 12: PE ruled out if D-dimer level is less than the age-adjusted cutoff value; (4) high probability of PE if 4PEPS is greater than 12: PE ruled out by imaging without preceding D-dimer test. In the first and the second external validation cohorts, the area under the receiver operator characteristic curves were 0.79 (95% CI, 0.76 to 0.82) and 0.78 (95% CI, 0.74 to 0.81), respectively. The false-negative testing rates if the 4PEPS strategy had been applied were 0.71% (95% CI, 0.37 to 1.23) and 0.89% (95% CI, 0.53 to 1.49), respectively. The absolute reductions in imaging testing were −22% (95% CI, −26 to −19) and −19% (95% CI, −22 to −16) in the first and second external validation cohorts, respectively. The 4PEPS strategy compared favorably with all recent strategies in terms of imaging testing.

**Conclusions and Relevance:**

The 4PEPS strategy may lead to a substantial and safe reduction in imaging testing for patients with suspected PE. It should now be tested in a formal outcome study.

## Key Points

**Question:**

Can a pretest probability score make it possible to rule out pulmonary embolism solely on clinical criteria and optimized D-dimer measurement to safely decrease imaging testing?

**Findings:**

In this study, the 4-Level Pulmonary Embolism Clinical Probability Score (4PEPS) was derived and validated using databases from 3 merged management studies. The safety and the efficacy of the 4PEPS strategy was confirmed in 2 external validation cohorts (false-negative rates: 0.71% and 0.89%; absolute reductions in imaging testing: −19% and −22%, respectively).

**Meaning:**

The 4PEPS strategy may lead to a substantial and safe reduction in imaging testing for patients with suspected pulmonary embolism.

## Teaser

This study derives and validates a 4-level pretest probability rule that makes it possible to rule out pulmonary embolism solely on clinical criteria and optimized D-dimer measurement to safely decrease imaging testing for suspected pulmonary embolism.

## Introduction

Despite the significant progress of the last decades, diagnosing pulmonary embolism (PE) remains a clinical challenge. The standard diagnostic strategy, based on clinical probability assessment, D-dimer testing, and computed tomography pulmonary angiography (CTPA), is proven to have a very low rate of diagnostic failure.^1^ However, there has been a large increase in CTPA for suspected PE.^2,3^ The exact reasons are likely multifactorial. The signs and symptoms of PE are very common and unspecific. As such, clinicians fear they might be missing a life-threatening condition and are prone to initiate a diagnostic process. Due to the lack of specificity of D-dimer testing, a large proportion of patients have a false-positive result and require imaging to rule out PE. Finally, CTPA is readily available, fast, minimally invasive, and more sensitive than ventilation/perfusion (V/Q) scans. A slight increase in PE diagnosis has been observed as a result but with no clear benefits in terms of outcome, especially PE-related mortality.^4,5^ One explanation is that because more CTPAs are being performed, there is a greater risk of false-positive results or non–clinically relevant diagnoses. Moreover, CTPA exposes patients to risks of allergies, kidney failure, and cumulative radiation-induced cancer.^6,7^ Several strategies have therefore been proposed to reduce PE overtesting and overdiagnosis (Table 1).^4,8,9,10,11,12,13,14^ These have proved satisfactory in terms of safety and efficacy, but they are based on different methods of assessing clinical pretest probability (CPP; eg, Wells^9^ or revised Geneva^8^ scores for PE, Pulmonary Embolism Rule-out Criteria [PERC] strategy,^10^ or YEARS strategy^12^), thus making it difficult to combine them and increasing the risk of misuse in clinical practice.

**Table 1.** Diagnostic Strategy Aiming to Reduce Imaging Testing

PE strategy	No diagnostic test required	D-dimer test required	D-dimer cutoff value	CTPA or V/Q scan required
Standard	NA	Nonhigh CPP with RG score (0-10), Wells score (0-4), or gestalt^a^^,^^b^	<0.5 μg/mL	High CPP or positive D-dimer test result
PERC strategy^c^	Low CPP with gestalt and negative PERC score^4^ (0)	Low CPP and positive PERC score (>0) or intermediate CPP with gestalt	<0.5 μg/mL	High CPP or positive D-dimer test result
ADJUST-PE strategy^d^	NA	Nonhigh CPP with RG score^a^ (0-10) or Wells score (0-4)^a^	Age adjusted^d^	High CPP or positive D-dimer test result
YEARS strategy^e^	NA	YEARS score negative (0)^e^	<1.0 μg/mL	Positive D-dimer test result
		YEARS score positive (>0)	<0.5 μg/mL	
PEGeD strategy^f^	NA	Low CPP with Wells score (0-4)^b^	<1.0 μg/mL	High CPP or positive D-dimer test result
		Moderate CPP with Wells score (4.5-6)	<0.5 μg/mL	
4PEPS	Very low CPP with 4PEPS (<0)	Low CPP with 4PEPS (0-5)	<1.0 μg/mL	High CPP with 4PEPS (>12) or positive D-dimer test result
		Moderate CPP 4PEPS (6-12)	Age adjusted^g^	

Abbreviations: 4PEPS, 4-Level Pulmonary Embolism Clinical Probability Score; ADJUST-PE, Age-Adjusted D-Dimer Cutoff Levels to Rule Out Pulmonary Embolism; CPP, clinical pretest probability; CTPA, computed tomography pulmonary angiography; PE, pulmonary embolism; PEGeD, Pulmonary Embolism Graduated d-Dimer; PERC, Pulmonary Embolism Rule-out Criteria; RG, revised Geneva; V/Q, ventilation/perfusion.

SI conversion factor: To convert D-dimer to nanomoles per liter, multiply by 5.476.

^a^ RG score: age of 65 years or older (+1), previous deep venous thrombosis or PE (+3), surgery or lower limb fracture in the past month (+2), active cancer (+2), unilateral lower limb pain (+3), hemoptysis (+2), heart rate of 75 to 94 beats per minute (+3) or 95 beats per minute or greater (+5), pain on lower limb deep venous palpation and unilateral edema (+4).^8^

^b^ Wells score (revised Wells score for PE): active cancer (+1), surgery or bedridden for 3 or more days during the past 4 weeks (+1.5), previous deep venous thrombosis or PE (+1.5), hemoptysis (+1), heart rate greater than 100 beats per minute (+1.5), clinical signs of deep venous thrombosis (+3), PE is the most likely diagnosis (+3).^9^

^c^ PERC strategy: age of 50 years or older (+1), heart rate of 100 beats per minute or greater (+1), room air pulse oximetry less than 95% (+1), unilateral leg edema (+1), hemoptysis (+1), recent surgery or trauma in the past 4 weeks (+1).^10^

^d^ ADJUST-PE strategy study: age-adjusted D-dimer cutoff value less than 0.5 μg/mL for patients younger than 50 years and calculated as age × 0.01 μg/mL for patients 50 years or older.^11^

^e^ YEARS strategy: 3-factor clinical rule derived from revised Wells score for PE, including clinical signs of deep vein thrombosis (+1), hemoptysis (+1), and PE is the most likely diagnosis (+1).^12^

^f^ PEGeD strategy: strategy using the 3-level revised Wells score for PE.^13^

^g^ Age-adjusted cutoff value less than 0.5 μg/mL for patients younger than 50 years and calculated as age × 0.01 μg/mL for patients 50 years or older.

Our primary aim was to develop and validate a pretest probability score to safely reduce imaging testing by integrating all the previously proposed strategies: the 4-Level Pulmonary Embolism Clinical Probability Score (4PEPS). Our secondary goal was to retrospectively assess the safety of a diagnostic strategy based on this new score and its efficacy in reducing imaging testing.

## Methods

### Study Design

Four levels of CPP for 4PEPS were defined a priori:

- Very low CPP, allowing exclusion of PE on clinical criteria only.
- Low CPP, allowing exclusion of PE with a high-sensitivity D-dimer level less than 1.0 μg/mL (to convert to nanomoles per liter, multiply by 5.476).
- Moderate CPP, allowing exclusion of PE with a D-dimer level less than 0.5 μg/mL or less than the age-adjusted cutoff value (calculated as age × 0.01 μg/mL for patients older than 50 years).
- High CPP, not allowing a safe exclusion of PE with D-dimer testing and requiring imaging testing (CTPA or V/Q scan).

To derive the score, we predefined the upper limit for PE prevalence in each CPP category using the bayesian approach and considered 2% as the safety threshold for PE.^13,15,16^ The negative likelihood ratios of a D-dimer test using 1.0 μg/mL as the cutoff value and using an age-adjusted cutoff value were established using the results of the YEARS study^12^ and the Age-Adjusted D-Dimer Cutoff Levels to Rule Out Pulmonary Embolism (ADJUST-PE) study.^11^ They were found to be 0.08 and 0.01, respectively. Accordingly, to achieve a posttest probability less than 2%, the upper limit of PE prevalence was set at 20% for low CPP and at 65% for moderate CPP. The present study was a retrospective analysis of data prospectively collected in 5 studies that were all approved by an ethical committee and performed with the informed consent of the participating patients. According to the current European legislation, an approval of an ethical committee was not required for the present study.

### Source of Data

For the derivation and internal validation, we merged 3 prospectively collected databases from patients with suspected PE (n = 11 114). The first study was performed in 117 emergency departments (EDs) in France and Belgium (n = 1529; enrolled in 2003)^17^; the second study was performed in 20 French EDs (n = 1645; enrolled in 2005 to 2006)^18^; and the third study was performed in 12 EDs in the US (n = 7940; enrolled in 2003 to 2006).^15^ Each database was randomly split into 2 groups, including 60% for the derivation cohort and 40% for the internal validation cohort.

Two other databases were used for external validation. The first study was performed in 6 EDs in France, Belgium, and Switzerland (n = 1819; enrolled in 2005 to 2006)^19^ and the second in 12 EDs in France and Belgium (n = 1757; enrolled in 2015 to 2016).^20^

### Outcome

The outcome was a PE diagnosed on CTPA or high-probability V/Q scan during the initial diagnostic workup or a venous thromboembolism (VTE) occurring during follow-up (3 months for the 4 European studies and 45 days for the US study) in a patient in whom PE was initially ruled out. In all studies, the following were considered as VTE: symptomatic PE objectively confirmed with CTPA or high-probability V/Q scan and/or deep vein thrombosis on compression ultrasonography and/or sudden unexpected death potentially related to PE according to an independent adjudication committee.

### 4PEPS Derivation

We evaluated all of the clinical variables known to be potentially associated with PE and available in the database.^21^ As patients were suspected of PE because of dyspnea or chest pain, these variables were not included. However, we took the variable of dyspnea and chest pain into account when both were present in a given patient. Variables with more than 2% of missing data were excluded, except those included in other prediction rules (PERC strategy,^10^ revised Geneva score,^8^ and Wells score^9^). Namely, the following variables were excluded: history of hypertension, diabetes, dyslipidemia, coronary disease, long travel, chronic kidney failure, smoking, family history of VTE, body weight, respiratory rate, and antiplatelet treatment. We categorized the continuous variables according to the cutoff values previously chosen in other scoring systems and according to their clinical relevance. There were 4 categories for age (younger than 50 years, aged 50 to 64 years, aged 65 to 80 years, and older than 80 years), 3 categories for heart rate (less than 80 beats per minute, 80 to 100 beats per minute, and more than 100 beats per minute) and temperature (less than 38 °C, 38 to 39 °C, and greater than 39 °C), and 2 categories for systolic blood pressure (less than 90 mm Hg and 90 mm Hg or greater) and pulse oximetry (Spo_2_; less than 95% and 95% or greater).

To select the predictor variables associated with PE, we performed a univariate analysis by using the χ^2^ test.^22^ All variables with a 2-tailed *P *value less than .20 as well as the nonsignificant variables included in other prediction rules were included in a multivariate logistic regression model. We performed a stepwise backward analysis including 1 variable for every 10 VTE events.^23,24^ We then removed the nonsignificant variables, considering a 2-tailed *P *value less than .05 as significant. Only significant variables were left in the final score. We assigned points for the score according to the regression coefficients. Finally, we chose the cutoff values to achieve the predefined levels of PE prevalence in each CPP category.^25^

### 4PEPS Validation

The accuracy of the score was assessed by calculating the receiver operating characteristic curve and analyzing the area under the receiver operating characteristic curve (AUC). The AUC confidence interval was computed with the DeLong-DeLong method.^26^ Calibration was assessed with the Hosmer-Lemeshow goodness-of-fit statistic.^23^ A Brier score was also reported, summarizing the magnitude of error in the probability forecasts as between 0.0 and 1.0, where a perfectly calibrated model would score 0.0.

### 4PEPS Strategy Safety and Efficacy Assessment

The safety of the 4PEPS strategy was retrospectively assessed using the false-negative rate if the strategy had been applied in the 2 external validation cohorts. This is the rate of PE diagnoses during the initial diagnostic process or VTEs found during the 3-month follow-up among patients with a very low CPP, a low CPP and D-dimer level less than 1.0 μg/mL, a moderate CPP and D-dimer level less than the age-adjusted cutoff value, or a negative CTPA or V/Q scan.

We defined the safety threshold of the 4PEPS strategy as a function of PE prevalence applying the recommendations of the International Society of Thrombosis and Hemostasis (1.82 + [0.00528 × prevalence]).^16^ The respective PE prevalences in the first and second external validation cohorts were 21.4% and 11.7%, respectively. Thus, the acceptable upper limits of the 95% CI of false-negative rates were predefined at 1.93% and 1.88%, respectively.^16^

Finally, the efficacy of the 4PEPS strategy was assessed by the rate of D-dimer and imaging testing, mainly CTPA, that could have been avoided if the 4PEPS strategy had been applied compared with the standard strategy, the PERC strategy,^10^ the ADJUST-PE strategy,^11^ the YEARS strategy,^12^ and the Pulmonary Embolism Graduated D-Dimer (PEGeD) strategy^13^ (Table 1).

### Missing Data

Analyses were performed including all analyzable patients. Patients with missing data were excluded and no imputation was performed. However, a sensitivity analysis was carried out for the 2 external validation cohorts considering the missing variables of 4PEPS as negative, ie, resulting in the lowest score and so representing the highest risk of a false-negative finding using the 4PEPS strategy.

### Statistical Analysis

We calculated the 95% CIs by using the Mid-*P* exact value performed using OpenEpi version 2, an open-source calculator. All other statistical analyses were performed using SPSS version 25.0 (SPSS Inc).

## Results

After exclusion of patients with missing data, 5588 patients were included in the derivation cohort (PE prevalence, 11.0%), 3726 in the internal validation cohort (PE prevalence, 11.7%), 1548 in the first external validation cohort (PE prevalence, 21.5), and 1669 in the second external validation cohort (PE prevalence, 11.7%). Of the 5588 patients in the derivation cohort, 3441 (61.8%) were female, and the mean (SD) age was 52 (18.5) years. In the 3 validation cohorts, 2265 of 3726 (60.7%), 842 of 1548 (54.4%), and 970 of 1669 (58.1%) were female, and the mean (SD) age was 52 (18.5), 59 (18.7), and 53 (19.8) years, respectively. Characteristics of the study samples are presented in Table 2.

**Table 2.** Baseline Characteristics of the Patients in the Different Cohorts

Characteristic	Cohort, No. (%)			
	Derivation (n = 5588)	Internal validation (n = 3726)	External validation	
			High prevalence (n = 1548)	Moderate prevalence (n = 1669)
Demographic characteristics				
Age, mean (SD), y	52 (18.5)	52 (18.5)	59 (18.7)	53 (19.8)
Male	2147 (38.4)	1461 (39.2)	706 (45.6)	699 (41.9)
Treatment and medical history				
Hormonal estrogenic treatment	417 (7.5)	272 (7.3)	132 (8.5)	189 (11.3)
History of VTE	705 (12.6)	486 (13.0)	266 (17.2)	199 (11.9)
Current malignancy^a^	688 (12.3)	403 (10.8)	114 (7.4)	133 (8.0)
Chronic respiratory disease	1121 (20.1)	724 (19.4)	193 (12.5)	139 (8.3)
Chronic heart failure	532 (9.5)	330 (8.9)	82 (5.3)	94 (5.6)
Immobility within 4 wk^b^	819 (14.7)	523 (14.0)	225 (14.6)	200 (12.0)
Pregnancy	83 (1.5)	61 (1.6)	0	15 (0.9)
Postpartum within 4 wk	84 (1.5)	48 (1.3)	12 (0.8)	9 (0.5)
Symptoms				
Chest pain	3572 (63.9)	2363 (63.4)	1070 (69.1)	1103 (66.1)
Dyspnea	3809 (68.2)	2545 (68.3)	1108 (71.6)	927 (55.5)
Chest pain and dyspnea	2323 (41.6)	1570 (42.1)	704 (45.5)	479 (28.7)
Syncope	496 (8.9)	328 (8.8)	321 (20.7)	315 (18.9)
Clinically suspected DVT^c^	620 (11.1)	403 (10.8)	270 (17.4)	242 (14.5)
Hemoptysis	187 (3.4)	123 (3.3)	71 (4.6)	47 (2.8)
Signs, mean (SD)				
Heart rate, beats per minute	92 (21.3)	92 (20.8)	8.7 (19.8)	87 (19.5)
Systolic blood pressure, mm Hg	133 (24.6)	133 (24.9)	139 (22.4)	136 (21.2)
Room air pulse oximetry, %	96 (4.7)	96 (4.4)	95 (5.0)	96 (3.7)
Temperature, °C	36.8 (0.7)	36.8 (0.7)	37.1 (1.3)	36.8 (0.7)
PE is the most likely diagnosis	1169 (20.9)	774 (20.7)	718 (46.4)	348 (20.9)
Final PE prevalence^d^	615 (11.0)	432 (11.6)	332 (21.5)	196 (11.7)

Abbreviations: DVT, deep vein thrombosis; PE, pulmonary embolism; VTE, venous thromboembolism.

^a^ Cancer or treatment for cancer within 1 year.

^b^ Surgery, lower limb plaster cast, or bedridden more than 3 days for acute medical condition within the last 4 weeks.

^c^ Unilateral lower limb spontaneous pain, pain on deep vein palpation, or swelling.

^d^ PE diagnosed during the initial diagnostic workup or symptomatic VTE occurred during the follow-up.

### 4PEPS Derivation

A univariate analysis found a statistical association with PE diagnosis for 21 variables. All of these were included in the multivariate regression. In addition, we included the variable of estrogenic treatment since this criterion is present in the PERC strategy.^10^ In the multivariate model, age of 65 to 80 years or older than 80 years, pulse rate of 80 to 100 beats per minute, systolic arterial pressure, hemoptysis, cancer, chronic cardiac failure, and pregnancy or post partum were not independently associated with PE. The remaining 13 variables were included in the final model, and we assigned points for each of them according to their regression coefficient. Table 3 represents the final model (4PEPS).

**Table 3.** 4-Level Pulmonary Embolism Clinical Probability Score (4PEPS)

Variable	Regression coefficient	Points
Age, y		
<50	−0.993	−2
50-64	−0.656	−1
Chronic respiratory disease	−0.570	−1
Heart rate <80 beats per minute	−0.406	−1
Chest pain and acute dyspnea	0.297	1
Male	0.472	2
Hormonal estrogenic treatment	0.608	2
Personal history of VTE	0.711	2
Syncope	0.504	2
Immobility within the last 4 wk^a^	0.509	2
Pulse oxygen saturation <95%	0.832	3
Calf pain and/or unilateral lower limb edema	1.009	3
PE is the most likely diagnosis	1.860	5
Clinical probability, total		
Very low CPP (<2%): PE can be ruled out	<0	
Low CPP (2%-20%): PE can be ruled out if D-dimer level <1.0 μg/mL	0-5	
Moderate CPP (20%-65%): PE can be ruled out if D-dimer level <0.5 μg/mL or <(age × 0.01) μg/mL	6-12	
High CPP (>65%): PE cannot be ruled out without imaging testing	≥13	

Abbreviations: CPP, clinical pretest probability; PE, pulmonary embolism; VTE, venous thromboembolism.

SI conversion factor: To convert D-dimer to nanomoles per liter, multiply by 5.476.

^a^ Surgery, lower limb plaster cast, or bedridden more than 3 days for acute medical condition within the last 4 weeks.

The PE prevalence by 4PEPS and the distribution of 4PEPS in the derivation cohort are presented in the Figure and the eTable in the Supplement. According to the predefined cutoff values, a 4PEPS less than 0 corresponds to a very low CPP (less than 2%), a 4PEPS of 0 to 5 corresponds to a low CPP (less than 20%), a 4PEPS of 6 to 12 corresponds to a moderate CPP (less than 65%), and a 4PEPS greater than 12 corresponds to a high CPP (65% or greater) (Table 3). PE prevalence in the very low category was 1.1% (95% CI, 0.6-1.6); low category, 6.2% (95% CI, 5.3-7.1); intermediate category, 31.3% (95% CI, 28.6-34.1); and high category, 73.6% (95% CI, 65.2-82.0).

**Figure.**
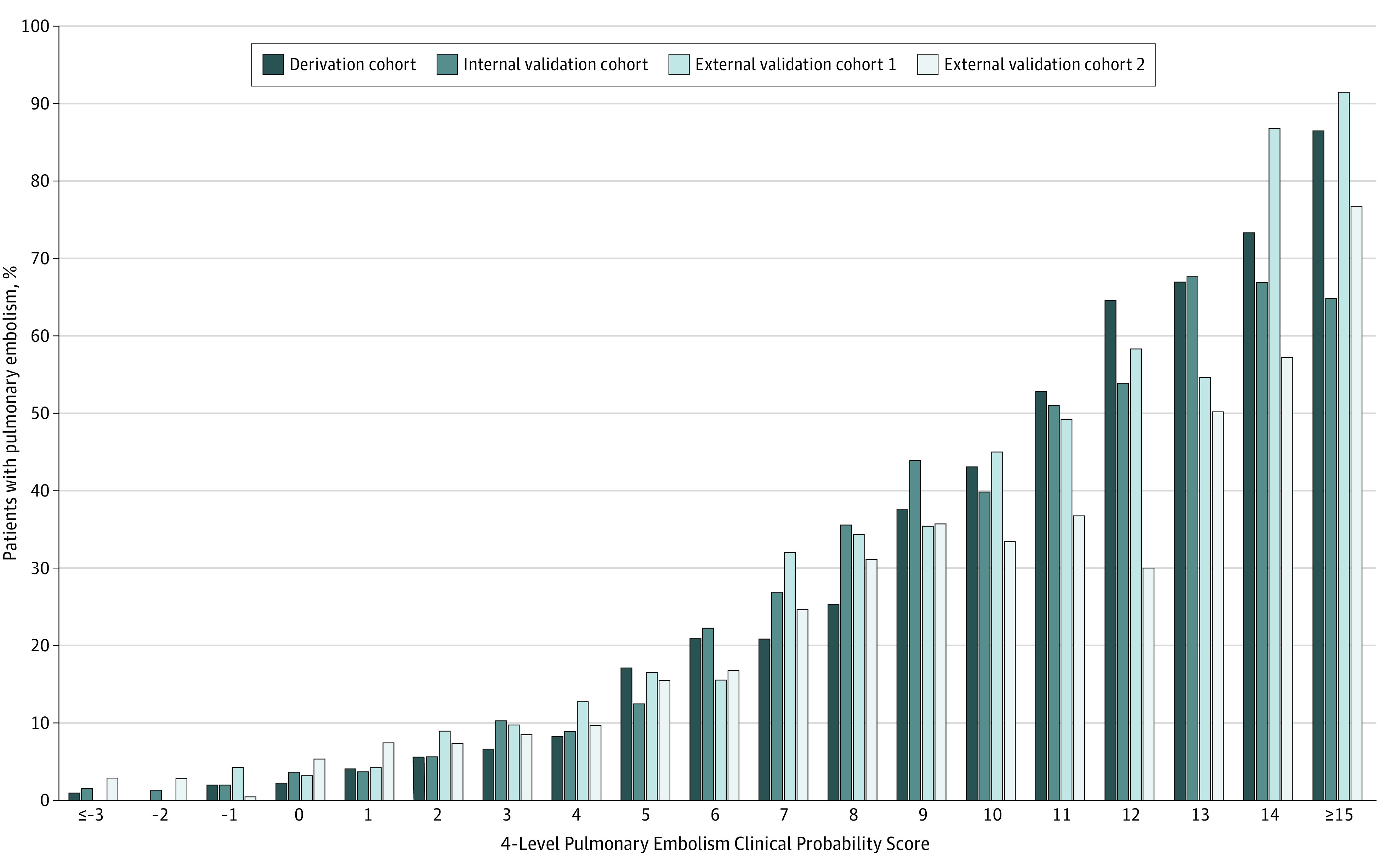
Pulmonary Embolism Prevalence by 4-Level Pulmonary Embolism Clinical Probability Score (4PEPS) in the Derivation and Validation Cohorts

### 4PEPS Validation

For the 3 validation cohorts, the PE prevalence by 4PEPS and the distribution of the 4PEPS are presented in the Figure and the eTable in the Supplement. In the internal validation cohort, the AUC was 0.83 (95% CI, 0.81-0.85). In the first and second external validation cohort, the AUCs were 0.79 (95% CI, 0.76-0.82) and 0.78 (95% CI, 0.74-0.81), respectively. The AUCs and the degree of concordance between the observed and predicted prevalence are presented in the eFigure in the Supplement.

### 4PEPS Strategy Validation

When the 4PEPS strategy was retrospectively applied in the first and second external validation cohorts, the false-negative rates were 11 of 1548 (0.71%; 95% CI, 0.37-1.23) and 14 of 1570 (0.89%; 95% CI, 0.53-1.49), respectively. No fatal PE or high-risk hemodynamically unstable PE were observed, and 3 of 11 false-negative VTEs in the high-prevalence cohort and 3 of 14 false-negative VTEs in the moderate-prevalence cohort were subsegmental PE. The upper limit of the 95% CI of the false-negative rate was less than the predefined cutoff value to consider the 4PEPS strategy as safe in the first (1.93%) and second (1.88%) external validation cohorts. Similar results were observed in the sensitivity analyses considering missing variables of 4PEPS as negative (high-prevalence cohort: 11 of 1687; false-negative rate, 0.65%; 95% CI, 0.34-1.13; moderate-prevalence cohort: 14 of 1655; false-negative rate, 0.85%; 95% CI, 0.50-1.61).

Compared with the standard strategy (CPP assessed using the revised Geneva score, D-dimer measurement with 0.5 μg/mL as the cutoff value), applying 4PEPS would have decreased the CTPA rate (external validation cohort 1: 46% vs 68%; difference, −22%; 95% CI, −26 to −19; external validation cohort 2: 32% vs 51%; difference, −19%; 95% CI, −22 to −16). Table 4^10,11,12,13^ compares the different strategies proposed to reduce diagnostic testing.

**Table 4.** Diagnostic Tests and False-Negative Testing According to the Strategy Retrospectively Applied

Strategy	External validation cohort, No. (%)					
	High prevalence (n = 1546)			Moderate prevalence (n = 1555)		
	D-dimer test	CTPA or V/Q scan	False-negatives	D-dimer test	CTPA or V/Q scan	False-negatives
Standard	1474 (95.3)	1058 (68.4)	4 (0.2)	1517 (97.6)	795 (51.1)	0
PERC strategy^10^	1188 (76.7)	981 (63.4)	16 (1.0)	1143 (73.5)	758 (48.7)	4 (0.3)
ADJUST-PE strategy^11^	1474 (95.3)	890 (57.6)	5 (0.3)	1517 (97.6)	714 (45.9)	0
YEARS strategy^12^	1546 (100)	885 (58.2)	11 (0.7)	1555 (100)	582 (37.4)	9 (0.6)
PEGeD strategy^13^	1429 (92.4)	817 (52.9)	12 (0.8)	1485 (95.5)	553 (35.6)	11 (0.7)
4PEPS	1341 (86.7)	713 (46.1)	11 (0.7)	1198 (77.0)	499 (32.1)	14 (0.9)

Abbreviations: 4PEPS, 4-Level Pulmonary Embolism Clinical Probability Score; ADJUST-PE, Age-Adjusted D-Dimer Cutoff Levels to Rule Out Pulmonary Embolism; CTPA, computed tomography pulmonary angiography; PEGeD, Pulmonary Embolism Graduated d-Dimer; PERC, Pulmonary Embolism Rule-out Criteria; V/Q, ventilation/perfusion.

## Discussion

Using 5 multicenter cohorts regrouping more than 12 000 patients suspected of PE, we were able to derive and validate a new clinical probability score to help physicians diagnose PE and safely decrease diagnostic imaging. Applying the 4PEPS diagnostic strategy retrospectively to 2 external validation cohorts, the rate of false-negative tests was below 1%, and the 4PEPS strategy performed better than all previously proposed strategies in terms of reducing imaging testing.

Overuse of CTPA for suspected PE is an important concern.^3^ There is increasing evidence that CTPA is frequently used inappropriately in patients for whom the benefits (probability of PE diagnosis and avoiding a PE complication) are outweighed by the risks (probability of a false-positive result, complication of anticoagulation, short-term or long-term adverse effect of CTPA).^2,3,27^ The first strategy developed to deal with overtesting was the PERC strategy.^10,15^ This can be used for patients for whom the clinician has already established a low clinical probability of PE based on an implicit gestalt impression. A negative PERC strategy finding defines a subgroup of these patients with a very low PE prevalence (less than 2%) allowing PE to be ruled out without any testing.^15^ However, applied alone or in association with the revised Geneva score, the PERC strategy appears to be insufficiently reliable.^28,29^ The 4PEPS strategy may not have such a limitation.

Another means to limit CTPA overuse is to optimize D-dimer testing. The ADJUST-PE study^11^ prospectively confirmed the safety and utility of an age-adjusted cutoff value for patients 50 years or older (Table 1). However, the effect of the ADJUST-PE strategy on imaging testing rates remains limited (−10.8% or −5.2% in our high-prevalence and moderate-prevalence external validation cohorts, respectively), particularly in young patients. A further proposal, based on the Bayes theorem, is to adjust the D-dimer cutoff value to the pretest probability.^30^ This principle was assessed in 2 recent studies, the YEARS study^12^ and PEGeD study.^13^ Both studies used 1.0 μg/mL as the D-dimer cutoff value for patients with a low CPP, and both achieved a very low overall rate of false-negative testing. Of note, the PEGeD study was the most recent study and has the lowest PE prevalence (7.4%), with 87% of patients having a low CPP.^13^ It should be used with caution in a population of patients with a higher PE prevalence. Indeed, recent external validation data of the PEGeD and YEARS strategies in cohorts of European patients suggest a higher failure rate.^31^ Moreover, since the methods of CPP assessment are different in the PERC strategy from the other strategies aiming to reduce overtesting, it is difficult to combine them.^10,11,12,13^ For example, to combine the PERC and PEGeD strategies, the physician may have to first assess implicit clinical probability (gestalt); second, if low, the PERC strategy; and third, if positive, the revised Wells score.^10,13^ The risk of misuse in clinical practice appears to be major and may have an important impact on safety. For example, although combining clinical gestalt and the PERC strategy has proven to be safe, the rate of failure when combining a low revised Geneva score and a negative PERC strategy finding is higher than 5%.^28^ Here lies the main benefit of 4PEPS: a single rule to guide diagnostic strategy resulting in a substantial reduction in testing, especially imaging testing.

Most of the 4PEPS criteria are included in other rules or scores for CPP assessment. Nevertheless, in our study, some potentially relevant criteria were not statistically associated with a PE diagnosis (pregnancy, history of cancer, chronic respiratory disease, hemoptysis). As the derivation database was large (n = 5588), we do not think that this is caused by a lack of power. More probably, we suppose that this result reflects the fact that physicians suspect PE at a very low threshold in patients with these characteristics.^32^ The first stage of the diagnostic process is deciding whether to investigate PE or not. This is why the PERC strategy needs to be combined with gestalt and why 4PEPS integrates the item PE is the most likely diagnosis. This criterion is sometimes criticized for a lack of objectivity and reproducibility. Nevertheless, it is included in the Wells score^9^ and YEARS strategy,^12^ is well-known by the ED physicians, and is easier to explain and to use than gestalt. The inclusion of factors decreasing the probability of PE diagnosis as well as factors increasing it allowed us to derive a 4-level score that rules out PE when negative. The 4PEPS calibration and accuracy of the 4PEPS appear to be at least similar to previous CPP scores for PE (Table 4).^8,33^

To facilitate 4PEPS implementation in clinical practice, an internet-application for smartphone and computer has been developed (https://peps.shinyapps.io/PEPS/). 4PEPS will be also incorporated in the new version of the decision-support software SPEED (Suspected Pulmonary Embolism in Emergency Departments; http://www.thrombus.fr/). We have previously shown that, compared with posters and pocket cards, such decision-support systems available on smartphones improves diagnostic decision-making and reduces the number of tests to reach a validated diagnostic decision.^18^ 4PEPS could also be integrated in the electronic medical record for automated calculation. Using such setups, we believe that 4PEPS will be embraced by ED physicians and will lead to a substantial and safe decrease in imaging testing.

### Strengths and Limitations

Our study has several strengths. We used a bayesian evidence-based medicine approach to define the prevalence limit in each CPP category, based on the predefined safety threshold and on the negative likelihood ratio of D-dimer.^34^ We followed a well-validated method to derive and validate the score and the recent recommendations of the International Society of Thrombosis and Hemostasis to assess the safety of the 4PEPS strategy in ruling out PE.^16,22^ The 5 databases of prospective multicenter international studies made it possible to define a large derivation cohort, an internal validation cohort, and 2 external validation cohorts. The results in terms of calibration and accuracy were very similar to each other, with an AUC around 80%. Finally, the safety of the 4PEPS strategy was confirmed in an external validation cohort with a moderate PE prevalence (11.7%) as well as in an external validation cohort with a high PE prevalence (21.5%). This reinforces the generalizability of our results.

Nevertheless, our study has some limitations. The studies used to derive and validate 4PEPS were all performed in ED settings and so 4PEPS may be not suitable for inpatients. Some variables were not systematically collected in these studies. They could not be included in our analyses. We also did not include patients with missing variables. However, the population for each cohort remains large, and similar results were obtained in the sensitivity analyses considering missing 4PEPS variables as negative. The score comprises 13 criteria that may be difficult to memorize, reinforcing the usefulness of an application for computer or handheld devices. Additionally, although we used clinical data from several prospective studies, we calculated this new score retrospectively. The 4PEPS strategy needs to be formally validated in a prospective implementation study.

## Conclusions

In conclusion, using a bayesian approach, we derived a new 4-level clinical probability score (4PEPS) to help ED physicians make decisions regarding patients suspected of PE. The accuracy, safety, and efficacy of the 4PEPS strategy were confirmed in 2 independent external validation cohorts, one with a moderate PE prevalence and the other with a high PE prevalence. For both cohorts, applying 4PEPS resulted in a very low rate of diagnostic failure and a substantial reduction in imaging testing. It should now be tested in a formal outcome study.

## Notes

Supplement. **eTable.** Distribution of 4PEPS among patients and patients with PE in derivation and validation cohorts. **eFigure.** Receiver operating characteristic curves and calibration plots.

## References

[1] Konstantinides SV, Meyer G, Becattini C, ; ESC Scientific Document Group. 2019 ESC guidelines for the diagnosis and management of acute pulmonary embolism developed in collaboration with the European Respiratory Society (ERS). Eur Heart J. 2020;41(4):543-603. doi:10.1093/eurheartj/ehz405 31504429

[2] Kline JA, Garrett JS, Sarmiento EJ, Strachan CC, Courtney DM. Over-testing for suspected pulmonary embolism in American emergency departments: the continuing epidemic. Circ Cardiovasc Qual Outcomes. 2020;13(1):e005753. doi:10.1161/CIRCOUTCOMES.119.005753 31957477

[3] Wang RC, Miglioretti DL, Marlow EC, . Trends in imaging for suspected pulmonary embolism across US health care systems, 2004 to 2016. JAMA Netw Open. 2020;3(11):e2026930. doi:10.1001/jamanetworkopen.2020.26930 33216141 PMC7679949

[4] Dobler CC. Overdiagnosis of pulmonary embolism: definition, causes and implications. Breathe (Sheff). 2019;15(1):46-53. doi:10.1183/20734735.0339-2018 30838059 PMC6395986

[5] Wiener RS, Schwartz LM, Woloshin S. Time trends in pulmonary embolism in the United States: evidence of overdiagnosis. Arch Intern Med. 2011;171(9):831-837. doi:10.1001/archinternmed.2011.178 21555660 PMC3140219

[6] Mitchell AM, Jones AE, Tumlin JA, Kline JA. Prospective study of the incidence of contrast-induced nephropathy among patients evaluated for pulmonary embolism by contrast-enhanced computed tomography. Acad Emerg Med. 2012;19(6):618-625. doi:10.1111/j.1553-2712.2012.01374.x 22687176 PMC5366244

[7] Niemann T, Zbinden I, Roser HW, Bremerich J, Remy-Jardin M, Bongartz G. Computed tomography for pulmonary embolism: assessment of a 1-year cohort and estimated cancer risk associated with diagnostic irradiation. Acta Radiol. 2013;54(7):778-784. doi:10.1177/0284185113485069 23761544

[8] Le Gal G, Righini M, Roy P-M, . Prediction of pulmonary embolism in the emergency department: the revised Geneva score. Ann Intern Med. 2006;144(3):165-171. doi:10.7326/0003-4819-144-3-200602070-00004 16461960

[9] Kearon C, Ginsberg JS, Douketis J, ; Canadian Pulmonary Embolism Diagnosis Study (CANPEDS) Group. An evaluation of D-dimer in the diagnosis of pulmonary embolism: a randomized trial. Ann Intern Med. 2006;144(11):812-821. doi:10.7326/0003-4819-144-11-200606060-00007 16754923

[10] Kline JA, Mitchell AM, Kabrhel C, Richman PB, Courtney DM. Clinical criteria to prevent unnecessary diagnostic testing in emergency department patients with suspected pulmonary embolism. J Thromb Haemost. 2004;2(8):1247-1255. doi:10.1111/j.1538-7836.2004.00790.x 15304025

[11] Righini M, Van Es J, Den Exter PL, . Age-adjusted D-dimer cutoff levels to rule out pulmonary embolism: the ADJUST-PE study. JAMA. 2014;311(11):1117-1124. doi:10.1001/jama.2014.2135 24643601

[12] van der Hulle T, Cheung WY, Kooij S, ; YEARS study group. Simplified diagnostic management of suspected pulmonary embolism (the YEARS study): a prospective, multicentre, cohort study. Lancet. 2017;390(10091):289-297. doi:10.1016/S0140-6736(17)30885-1 28549662

[13] Kearon C, de Wit K, Parpia S, ; PEGeD Study Investigators. Diagnosis of pulmonary embolism with D-dimer adjusted to clinical probability. N Engl J Med. 2019;381(22):2125-2134. doi:10.1056/NEJMoa1909159 31774957

[14] Singh B, Mommer SK, Erwin PJ, Mascarenhas SS, Parsaik AK. Pulmonary Embolism Rule-out Criteria (PERC) in pulmonary embolism—revisited: a systematic review and meta-analysis. Emerg Med J. 2013;30(9):701-706. doi:10.1136/emermed-2012-201730 23038695

[15] Kline JA, Courtney DM, Kabrhel C, . Prospective multicenter evaluation of the Pulmonary Embolism Rule-out Criteria. J Thromb Haemost. 2008;6(5):772-780. doi:10.1111/j.1538-7836.2008.02944.x 18318689

[16] Dronkers CEA, van der Hulle T, Le Gal G, ; Subcommittee on Predictive and Diagnostic Variables in Thrombotic Disease. Towards a tailored diagnostic standard for future diagnostic studies in pulmonary embolism: communication from the SSC of the ISTH. J Thromb Haemost. 2017;15(5):1040-1043. doi:10.1111/jth.13654 28296048

[17] Roy P-M, Meyer G, Vielle B, ; EMDEPU Study Group. Appropriateness of diagnostic management and outcomes of suspected pulmonary embolism. Ann Intern Med. 2006;144(3):157-164. doi:10.7326/0003-4819-144-3-200602070-00003 16461959

[18] Roy PM, Durieux P, Gillaizeau F, . A computerized handheld decision-support system to improve pulmonary embolism diagnosis: a randomized trial. Ann Intern Med. 2009;151(10):677-686. doi:10.7326/0003-4819-151-10-200911170-00003 19920268

[19] Righini M, Le Gal G, Aujesky D, . Diagnosis of pulmonary embolism by multidetector CT alone or combined with venous ultrasonography of the leg: a randomised non-inferiority trial. Lancet. 2008;371(9621):1343-1352. doi:10.1016/S0140-6736(08)60594-2 18424324

[20] Penaloza A, Soulié C, Moumneh T, . Pulmonary Embolism Rule-out Criteria (PERC) rule in European patients with low implicit clinical probability (PERCEPIC): a multicentre, prospective, observational study. Lancet Haematol. 2017;4(12):e615-e621. doi:10.1016/S2352-3026(17)30210-7 29150390

[21] West J, Goodacre S, Sampson F. The value of clinical features in the diagnosis of acute pulmonary embolism: systematic review and meta-analysis. QJM. 2007;100(12):763-769. doi:10.1093/qjmed/hcm113 18089542

[22] Wasson JH, Sox HC, Neff RK, Goldman L. Clinical prediction rules. applications and methodological standards. N Engl J Med. 1985;313(13):793-799. doi:10.1056/NEJM198509263131306 3897864

[23] Hosmer DW, Lemeshow S. Applied Logistic Regression. Wiley; 1989.

[24] Harrell FE Jr, Lee KL, Mark DB. Multivariable prognostic models: issues in developing models, evaluating assumptions and adequacy, and measuring and reducing errors. Stat Med. 1996;15(4):361-387. doi:10.1002/(SICI)1097-0258(19960229)15:4<361::AID-SIM168>3.0.CO;2-4 8668867

[25] Fagan TJ. Letter: nomogram for Bayes theorem. N Engl J Med. 1975;293(5):257. doi:10.1056/NEJM197507312930513 1143310

[26] DeLong ER, DeLong DM, Clarke-Pearson DL. Comparing the areas under two or more correlated receiver operating characteristic curves: a nonparametric approach. Biometrics. 1988;44(3):837-845. doi:10.2307/2531595 3203132

[27] Hutchinson BD, Navin P, Marom EM, Truong MT, Bruzzi JF. Overdiagnosis of pulmonary embolism by pulmonary CT angiography. AJR Am J Roentgenol. 2015;205(2):271-277. doi:10.2214/AJR.14.13938 26204274

[28] Penaloza A, Verschuren F, Dambrine S, Zech F, Thys F, Roy PM. Performance of the Pulmonary Embolism Rule-out Criteria (the PERC rule) combined with low clinical probability in high prevalence population. Thromb Res. 2012;129(5):e189-e193. doi:10.1016/j.thromres.2012.02.016 22424852

[29] Hugli O, Righini M, Le Gal G, . The Pulmonary Embolism Rule-out Criteria (PERC) rule does not safely exclude pulmonary embolism. J Thromb Haemost. 2011;9(2):300-304. doi:10.1111/j.1538-7836.2010.04147.x 21091866

[30] Kline JA, Hogg MM, Courtney DM, Miller CD, Jones AE, Smithline HA. D-dimer threshold increase with pretest probability unlikely for pulmonary embolism to decrease unnecessary computerized tomographic pulmonary angiography. J Thromb Haemost. 2012;10(4):572-581. doi:10.1111/j.1538-7836.2012.04647.x 22284935 PMC3319270

[31] Eddy M, Robert-Ebadi H, Richardson L, . External validation of the YEARS diagnostic algorithm for suspected pulmonary embolism. J Thromb Haemost. 2020. doi:10.1111/jth.15083 32869501

[32] Kline JA, Richardson DM, Than MP, Penaloza A, Roy PM. Systematic review and meta-analysis of pregnant patients investigated for suspected pulmonary embolism in the emergency department. Acad Emerg Med. 2014;21(9):949-959. doi:10.1111/acem.12471 25269575

[33] Penaloza A, Verschuren F, Meyer G, . Comparison of the unstructured clinician gestalt, the Wells score, and the revised Geneva score to estimate pretest probability for suspected pulmonary embolism. Ann Emerg Med. 2013;62(2):117-124.e2. 23433653 10.1016/j.annemergmed.2012.11.002

[34] Carpenter CR, Raja AS. Arming the bayesian physician to rule out pulmonary embolism: using evidence-based diagnostics to combat overtesting. Acad Emerg Med. 2014;21(9):1036-1038. doi:10.1111/acem.12450 25269585

