# Supplementary material for: Derivation and Validation of a 4-Level Clinical Pretest Probability Score for Suspected Pulmonary Embolism to Safely Decrease Imaging Testing
Source: JAMA Cardiol. 2021 Mar 3;6(6):1–9. doi: 10.1001/jamacardio.2021.0064 (PMC7931139; doi:10.1001/jamacardio.2021.0064)
Supplement: Supplement. — eTable. Distribution of 4PEPS among patients and patients with PE in derivation and validation cohorts. eFigure. Receiver operating characteristic curves and calibration plots. [file jamacardiol-e210064-s001.pdf]

Supplemental Online Content

Roy P-M, Friou E, Germeau B, et al. Derivation and validation of a 4-level clinical pretest probability score for suspected pulmonary embolism to safely decrease imaging testing. *JAMA Cardiol*. Published online March 3, 2021.
doi:10.1001/jamacardio.2021.0064

eTable. Distribution of 4PEPS among patients and patients with PE in derivation and validation cohorts

eFigure. Receiver operating characteristic curves and calibration plots

This supplementary material has been provided by the authors to give readers additional information about their work.

© 2021 Roy P-M et al. *JAMA Cardiology*.

eTable. Distribution of 4PEPS among patients and patients with PE in derivation and validation cohorts

	Derivation cohort (N=5588)		Internal validation (N=3726)		External validation 1 (N=1548)		External validation 2 (N=1669)	
4PEPS value	No.	no. with PE (%)	No.	no. with PE (%)	No.	no. with PE (%)	No.	no. with PE (%)
≤-3	227	2 (0.88)	137	2 (1.46)	15	0 (0.00)	34	1 (2.94)
-2	568	1 (0.18)	364	5 (1.37)	33	0 (0.00)	104	3 (2.88)
-1	660	13 (1.97)	445	9 (2.02)	70	3 (4.29)	209	1 (0.48)
0	745	17 (2.28)	484	18 (3.72)	93	3 (3.23)	265	14 (5.28)
1	650	26 (4.00)	466	18 (3.86)	118	5 (4.24)	215	16 (7.44)
2	402	23 (5.72)	314	18 (5.73)	121	11 (9.09)	136	10 (7.35)
3	415	27 (6.51)	252	26 (10.3)	123	12 (9.76)	128	11 (8.59)
4	408	34 (8.33)	289	26 (9.00)	156	20 (12.8)	103	10 (9.71)
5	328	56 (17.1)	216	27 (12.5)	151	25 (16.6)	116	18 (15.5)
6	281	59 (21.0)	176	39 (22.2)	142	22 (15.5)	65	11 (16.9)
7	198	41 (20.8)	138	37 (26.8)	100	32 (32.0)	73	18 (24.7)
8	198	50 (25.3)	107	38 (35.5)	96	33 (34.4)	45	14 (31.1)
9	139	52 (37.4)	105	46 (43.8)	96	34 (35.4)	56	20 (35.7)
10	116	50 (43.1)	68	27 (39.7)	69	31 (44.9)	45	15 (33.3)
11	74	39 (52.7)	57	29 (50.9)	61	30 (49.2)	30	11 (36.7)
12	73	47 (64.4)	39	21 (53.9)	43	25 (58.1)	20	6 (30.0)
13	51	34 (66.7)	37	25 (67.6)	23	12 (52.2)	8	4 (50.0)
14	26	19 (73.1)	15	10 (66.7)	15	13 (86.7)	7	4 (57.1)
≥15	29	25 (86.2)	17	11 (64.7)	23	21 (91.3)	10	9 (90.0)

© 2021 Roy P-M et al. *JAMA Cardiology*.

eFigure. Receiver operating characteristic curves and calibration plots

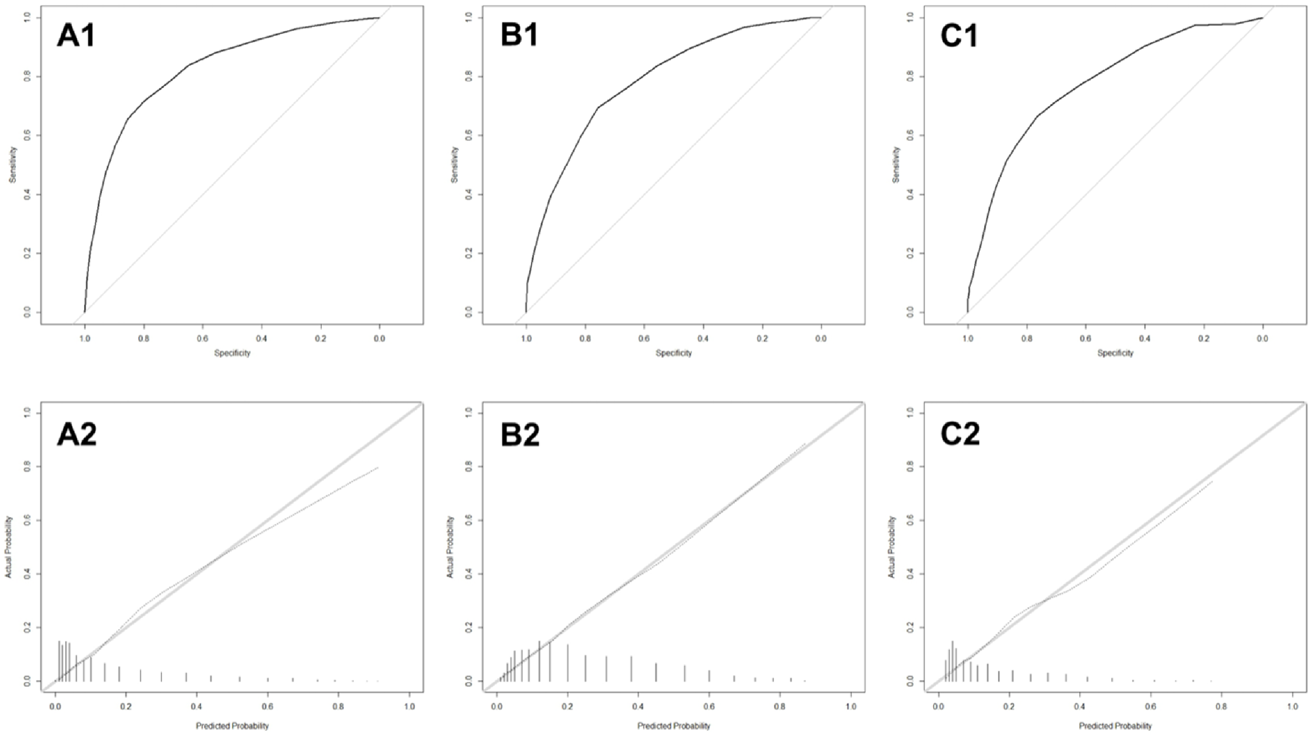

Receiver operating characteristic curve (Panels 1) and calibration plots (Panels 2) for 4PEPS in the internal validation cohort (Panels A), the first external validation cohort (Panels B) and the second external validation cohort (Panels C). For calibration plots (Panels 2), the grey zone represents the ideal distribution. The distributions of predicted probabilities are shown at the bottom of the graphs. The Hosmer and Lemeshow goodness of fit and Brier score were 0.753 and 0.082, 0.710 and 0.420, and, 0.134 and 0.089, in the internal validation cohort, the first and second external validation cohorts, respectively.

© 2021 Roy P-M et al. *JAMA Cardiology*.
